# 6,7-Dihalo-Benzothiadiazines as Potent and Selective AMPA Receptor Modulators for Cognitive Enhancement and Neuroprotection

**DOI:** 10.1021/acsmedchemlett.6c00014

**Published:** 2026-02-09

**Authors:** Yinlong Li, Hongjie Yuan, Steven H. Liang

**Affiliations:** † Department of Radiology and Imaging Sciences, 1371 Emory University, 1364 Clifton Road, Atlanta, Georgia 30322, United States; ‡ Department of Pharmacology and Chemical Biology, 12239 Emory University School of Medicine, Atlanta, Georgia 30322, United States; § Wallace H. Coulter Department of Biomedical Engineering, Georgia Institute of Technology and Emory University, Atlanta, Georgia 30332, United States

**Keywords:** AMPA receptor, Positive allosteric modulators, Structure−activity relationship, Neurological disorders

## Abstract

α-Amino-3-hydroxy-5-methyl-4-isoxazolepropionic acid (AMPA) receptors are tetrameric ionotropic glutamate receptors that mediate fast excitatory synaptic transmission in the brain and represent important therapeutic targets for neurological disorders. Positive allosteric modulators (PAMs) of AMPA receptors enhance rapid excitatory signaling by increasing receptor’s sensitivity to glutamate and have been widely explored as agents to improve cognitive function in central nervous system (CNS) diseases. Structural modification of 3,4-dihydro-2H-1,2,4-benzothiadiazine 1,1-dioxides (BTDs) analogs is a key strategy to develop potent AMPAR PAMs. A recent study reported a new pharmacomodulation strategy on the benzene ring of BTDs through systematic structure–activity relationship (SAR) optimization. This work led to the identification of compound **14o** (BPAM363), which exhibits improved pharmacological properties, robust *in vivo* cognitive-enhancing and neuroprotective effects. These findings provide valuable insight for further development of AMPAR PAMs as therapeutic candidates for cognitive disorders.

## Graphical abstract

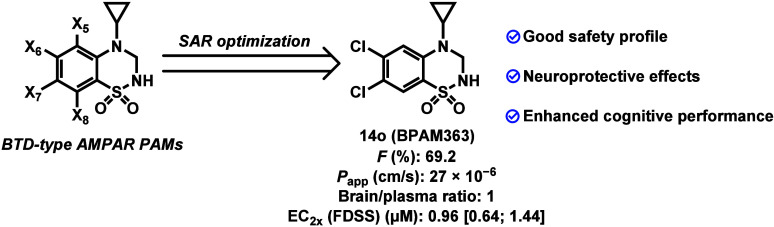

Ionotropic glutamate receptors (iGluRs) are a major class of ligand-gated ion channels that mediate excitatory neurotransmission in the central nervous system (CNS).^1–3^ iGluRs play key roles in synaptic plasticity, learning, and memory, and are categorized into three subfamilies based on pharmacology and sequence homology: α-amino-3-hydroxy-5-methyl-4-isoxazolepropionic acid (AMPA) receptors, *N*-methyl-d-aspartate (NMDA) receptors, and kainate receptors.^4,5^ Among these, AMPA receptors (AMPARs) mediate the majority of rapid excitatory signaling and are crucial for regulating synaptic strength and neuronal communication.^6,7^ AMPARs are tetrameric assemblies composed of combinations of four subunits (GluA1, GluA2, GluA3, and GluA4), with each contributing to unique channel gating, ion permeability, and trafficking.^8,9^ The endogenous agonist l-glutamate binds to the orthosteric site to trigger membrane depolarization and fast excitatory synaptic transmission.^10^ In contrast, allosteric modulators of AMPARs, including negative allosteric modulators (NAMs) and positive allosteric modulators (PAMs) modulate receptor ionic currents by targeting binding sites distinct from the glutamate orthosteric site.^11–13^ Specifically, AMPAR PAMs have been shown to facilitate long-term potentiation, and have demonstrated promise as therapeutic agents for cognitive and neurodegenerative disorders in both preclinical and clinical studies.^14,15^ Over the past decades, benzothiadiazine derivatives have emerged as one of the most widely investigated classes of AMPAR PAMs, and these modulators have substantially contributed to elucidating the regulatory mechanisms of AMPARs.^16–18^ 1,2,4-Benzothiadiazine 1,1-dioxides (BTDs) feature a heterocyclic core containing a sulfonyl group and two nitrogen atoms that mediate hydrogen-bonding and polar interactions within the AMPAR allosteric site. Subtle pharmacomodulations of the core scaffold or peripheral substituents can markedly influence their potency and subtype selectivity.^19,20^ As illustrated in Figure 1, structure-based optimization of alkyl substituents, halogenated and aromatic groups, has led to the identification of multiple sulfonamide-based AMPAR PAMs, including cyclothiazide,^21^ IDRA-21,^22^ BPAM344,^23^ BPAM521,^24^ BPAM395,^25^ BPAM279,^25^ S18986,^26^ and TAK-137.^27^ Although these compounds display micromolar-range potency and favorable pharmacological profiles, further structure–activity relationship (SAR) optimization of BTDs is needed to improve potency and maximize their translational potential.

**Figure 1:**
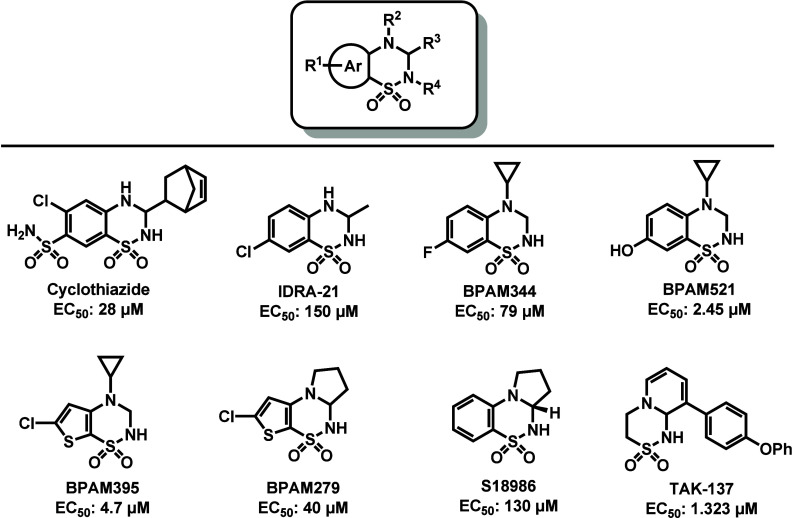
Representative benzothiadiazine-derived AMPAR PAMs.

Building on previous studies, Lesenfants et al. reported a systematic SAR investigation of BTD derivatives by modifying substituents on the benzene ring and varying the *N*-alkyl groups and evaluated their effects on cognitive enhancement and neuroprotective efficacy.^28^ They first investigated monohalo-substituted BTD derivatives on the benzene ring, and their potency was assessed using an *in vitro* fluorescence assay (FDSS) on primary cultures from rat embryonic cortex. The results showed that the activity was significantly influenced by halogen position (7 > 8 > 6 or 5) and type (*F* > Cl > Br). Next, they examined di­(fluoro/chloro)-substituted BTDs and found that halogenation at the 6,7-, 6,8-, or 7,8-positions produced potent modulators with EC_2*x*_ values below 1 μM, exemplified by compounds **14f**, **14h**, **14j**, **14l**, and **14o** (Table 1). Notably, the halogen at the 8-position in 7,8-dihalo derivatives (**14j**, **14l**) had little effect on activity, while a chlorine at the 7-position was preferred over fluorine. Considering that compound **14o** (BPAM363) exhibited the best safety profile at very high oral doses (100 mg/kg) in mice, it was selected for further *in vitro* and *in vivo* studies.

**Table 1:**
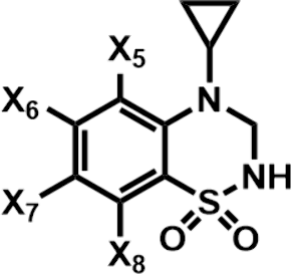
Effects of Di­(fluoro/chloro)-Substituted BTD Derivatives

Compound#	X_5_	X_6_	X_7_	X_8_	EC_2*x*_ (FDSS^a^) (μM)
**14f**	H	F	H	F	0.77 [0.30; 1.94] (*n* = 3)
**14h**	H	F	Cl	H	0.19 (*n* = 2)
**14j**	H	H	F	F	0.83 [0.70; 0.99] (*n* = 3)
**14l**	H	H	F	Cl	0.53 [0.41; 0.69] (*n* = 3)
**14o**	H	Cl	Cl	H	0.96 [0.64; 1.44] (*n* = 6)

a EC_2*x*_ is defined as the concentration of the modulator that produces a twofold increase in AMPA (300 μM) induced fluorescence, as measured using the FDSS fluorescence assay in primary rat brain cultures (*n* = 2–6). Data are reported as geometric means with the corresponding 95% confidence intervals shown in brackets when *n* > 2. The data was adapted from ref 28. Copyright 2025 American Chemical Society.

Compound **14o** exhibited a concentration-dependent potentiation of AMPA receptor-mediated currents in rat cortex mRNA-injected oocytes, reaching a maximal enhancement of approximately 26-fold relative to AMPA alone. In addition, compound **14o** displayed high selectivity for AMPA receptors, as no significant effect on NMDA- or kainate-induced currents was observed (Figure 2A). In hippocampal slices from Wistar rats, compound **14o** enhanced excitatory postsynaptic responses in the CA1 region in a concentration-dependent manner following Schaffer collateral stimulation, with significant increases in the area of the evoked postsynaptic response observed at concentrations of 10 and 30 μM. (Figure 2B). In primary cortical neuronal cultures, compound **14o** dose-dependently elevated the expression of brain-derived neurotrophic factor (BDNF), a key neurotrophin involved in synaptic plasticity (0.3–10 μM) (Figure 2C). To assess the cognitive effects, long-term potentiation (LTP) was evaluated in the dentate gyrus of the hippocampus in anesthetized Wistar rats. Compared with the vehicle-treated control, compound **14o** significantly enhanced both induction and maintenance of synaptic potentiation (Figure 2D). To evaluate effects of compound **14o** on working memory, a spontaneous alternation task in a T-maze was performed. Administration of compound **14o** at low doses (0.01 and 0.03 mg/kg, i.p.) significantly improved spontaneous alternation performance compared with vehicle-treated mice, consistent with enhanced working memory function (Figure 2E). Neuroprotective effects of compound **14o** were confirmed in a rat model of delayed hippocampal neuronal cell death. Treatment with compound **14o** (10 mg/kg, i.p.) significantly delayed hippocampal neuronal loss observed 7 days after transient global ischemia. Notably, compound **14o** preserved the viability of over 80% of hippocampal neurons, compared with only 31% viable cells in ischemic rats receiving vehicle treatment. This neuroprotective efficacy is higher than other known AMPA receptor PAMs, such as S18986 (Figure 2F). Collectively, these results highlight the therapeutic potential of compound **14o** as a promising neuroprotective candidate with the ability to delay neuronal degeneration and enhance cognitive function.

**Figure 2:**
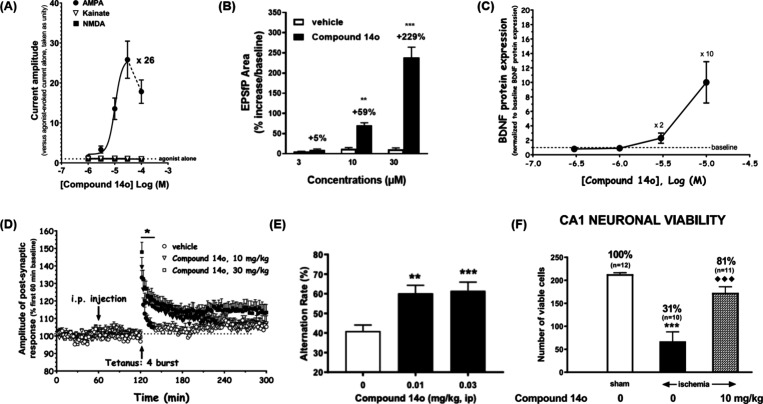
(A) Effect of compound **14o** on AMPA-, kainate-, or NMDA-induced currents in rat cortex mRNA-injected oocytes. (B) Effect of compound **14o** on AMPA-mediated postsynaptic responses in CA1 hippocampal slices (*n* = 5), expressed as% increase in response area. (C) Compound **14o** increases BDNF protein levels in rat primary cortical cultures, normalized to basal levels across varying concentrations. (D) Effect of compound **14o** on LTP of postsynaptic responses in the dentate gyrus evoked by tetanic stimulation of the perforant path in anesthetized rats (*n* = 8). (E) Effect of compound **14o** (0.01 and 0.03 mg/kg, i.p.) on T-maze spontaneous alternation in C57BL/6 mice (spatial working memory). (F) Effect of compound **14o** on CA1 hippocampal neuron viability, assessed 7 days after transient global cerebral ischemia in Wistar rats. The data was adapted from ref 28. Copyright 2025 American Chemical Society.

## Future Outlook

Benzothiadiazine derivatives have emerged as one of the most extensively studied classes of AMPAR PAMs. The medicinal chemistry team at the University of Liège conducted comprehensive SAR studies and have identified several potent candidates characterized by favorable safety profiles, high oral bioavailability, and effective CNS penetration. Although these advances have accelerated the translational potential of AMPAR PAMs, their clinical implementation remains a considerable challenge, highlighting the need for further structural optimization and exploration of alternative scaffolds to improve potency and selectivity while minimizing the risk of adverse effects. Importantly, emerging technologies such as positron emission tomography (PET) imaging targeting AMPA receptors can greatly facilitate the development of AMPAR PAMs by enabling *in vivo* target engagement and pharmacokinetic–pharmacodynamic (PK/PD) assessments.^29,30^ For example, the development of a ^18^F-labeled analog of BPAM344 ([^18^F]­AMPA-2109) demonstrates the feasibility of this approach, which shows good blood–brain barrier (BBB) permeability in rodents and nonhuman primates.^31^ These imaging tools provide critical insights and foundation for the rational design of the next generation of AMPAR-selective modulators and their successful transition into clinical application.

## Notes

No unexpected or unusually high safety hazards were encountered

## Abbreviations

AMPA: α-Amino-3-hydroxy-5-methyl-4-isoxazolepropionic acid
PAMs: Positive allosteric modulators
CNS: Central nervous system
BTDs: 3,4-Dihydro-2H-1,2,4-benzothiadiazine 1,1-dioxides
SAR: Structure–activity relationship
iGluRs: Ionotropic glutamate receptors
NMDA: *N*-Methyl-d-aspartate
AMPARs: AMPA receptors
NAMs: Negative allosteric modulators
FDSS: *In vitro* fluorescence assay
BDNF: Brain-derived neurotrophic factor
LTP: Long-term potentiation
PET: Positron emission tomography
BBB: Blood–brain barrier
